# Progesterone in recurrent pregnancy loss: from controversial efficacy to mechanism-based patient stratification

**DOI:** 10.3389/fphar.2026.1778288

**Published:** 2026-04-17

**Authors:** Chun-fei Wang, Yu-fei Zhang, Jin-ke Li, Xue-feng Jiao, Qiang Wei

**Affiliations:** 1 Department of Obstetrics and Gynecology, West China Second University Hospital, Sichuan University, Chengdu, China; 2 Key Laboratory of Birth Defects and Related Diseases of Women and Children (Sichuan University), Ministry of Education, Chengdu, China; 3 Department of Pharmacy/Evidence-Based Pharmacy Center, West China Second University Hospital, Sichuan University, Children’s Medicine Key Laboratory of Sichuan Province, Chengdu, China

**Keywords:** biomarkers, personalized therapy, precision medicine, progesterone, recurrent pregnancy loss

## Abstract

Progesterone supplementation has long been a controversial therapeutic intervention for recurrent pregnancy loss (RPL). Previous randomized controlled trials have yielded conflicting results, largely due to a “one-size-fits-all” approach that treats RPL as a homogeneous disease. From a pharmacological standpoint, this highlights the key challenge of patient heterogeneity in drug response. This review re-evaluates the role of progesterone by examining its different molecular mechanisms of action, including genomic and non-genomic signaling, immunomodulation (e.g., Treg cell induction, uterine natural killer cell regulation), and the modulation of endometrial receptivity. We then characterize the molecular heterogeneity of RPL, defining putative subtypes such as the “immune-dysregulated,” “receptivity-defective,” and “endocrine-insufficient” phenotypes. Crucially, we contend that the efficacy of progesterone is tightly linked to these specific pathological mechanisms. Finally, we propose a precision pharmacology framework that advocates for the use of mechanism-based biomarkers, such as endometrial transcriptomic signatures and immune cell profiles, to identify patient subgroups most likely to benefit from progesterone therapy. This paradigm shift from empirical supplementation to biomarker-guided prescription not only holds the potential to resolve long-standing controversies, but also paves the way for more effective, personalized pharmacotherapeutic strategies in RPL.

## 1 Introduction

Recurrent pregnancy loss (RPL), defined as the loss of two or more consecutive pregnancies before 20–24 weeks of gestation, affects 1%–5% of women of reproductive age worldwide (ESHRE Guideline Group on RPL et al., 2023; Rai and Regan, 2006). The etiology of RPL is highly complex (Toth et al., 2022), involving factors such as chromosomal or genetic abnormalities (Dong et al., 2019; Li et al., 2021), uterine anatomical defects (Kim et al., 2021), autoimmune diseases (Alijotas-Reig et al., 2020), thrombophilia (Eslami et al., 2020; Nahas et al., 2018), endocrine disorders (Andersen and Andersen, 2021; Pluchino et al., 2014), infections (Al-Memar et al., 2020; Kimura et al., 2019), male factors, and environmental or psychological influences (Quenby et al., 2021). Notably, a substantial proportion of RPL cases remain unexplained (URPL) after comprehensive evaluation, suggesting that RPL is a highly heterogeneous clinical syndrome rather than a single disease entity (de Assis et al., 2024).

In this context, progesterone, a key steroid hormone in establishing and maintaining pregnancy, has been widely studied for its therapeutic potential in RPL. It plays a vital role in endometrial receptivity, embryo implantation, and early pregnancy sustenance. For decades, progesterone has been commonly utilized as an empirical treatment for RPL. However, its clinical efficacy remains controversial. This controversy mainly stems from two core challenges: first, the significant heterogeneity within the patient population, including diverse etiologies, as well as variations in progesterone dosage, administration route, and treatment timing (Devall and Coomarasamy, 2020); and second, an incomplete understanding of its full range of mechanisms, particularly its functions beyond endometrial support (Shah et al., 2018).

Progestogens are broadly classified into two categories (Schindler et al., 2003). The first comprises natural progestogens, which are identical in structure to the progesterone secreted by the human body. Progesterone itself is the primary example, available in oral capsules, vaginal suppositories, and injectable formulations. Among them, oral progesterone capsules undergo extensive first-pass metabolic, markedly limiting their efficacy, and high doses may increase the risk of intrahepatic cholestasis in susceptible women. However, vaginal or intramuscular progesterone is not subject to this limitation, so it has become the main drug route choice (Bacq et al., 1997; Choavaratana and Manoch, 2004). The second category consists of synthetic progestins, which are chemically modified derivatives of natural progesterone, including compounds such as dydrogesterone, norethisterone and levonorgestrel. Among these, only natural progesterone and dydrogesterone are currently approved for clinical use during pregnancy. Compared to natural progesterone, dydrogesterone offers higher oral bioavailability and exhibits high selectivity for the progesterone receptor (PR), with minimal anti-androgenic activity at the pre-receptor level (Piette, 2018). This profile minimizes off-target receptor activation and associated adverse effects. Current evidence indicates that oral dydrogesterone provides comparable efficacy to vaginal micronized progesterone in luteal phase support, achieving similar pregnancy rates, while significantly reducing adverse effects such as perineal irritation, vaginal bleeding, increased discharge, and interference with sexual activity (Griesinger et al., 2019).

Recent breakthroughs in molecular biology and immunology have unveiled the pleiotropic mechanisms of progesterone. Beyond inducing secretory transformation of the endometrium to provide a suitable environment for implantation, progesterone also modulates maternal-fetal immune tolerance. It promotes a T helper 2 cell (Th2) cytokine bias (Wang et al., 2020), facilitates regulatory T cell (Treg) differentiation (Abdolmohammadi Vahid et al., 2019), inhibits natural killer cell cytotoxicity (Wilkens et al., 2013), supports uterine spiral artery remodeling, and suppresses uterine smooth muscle contractility (Maliqueo et al., 2016). These discoveries not only deepen our understanding of the function of progesterone but also provide a foundation for mechanism-based patient stratification. Such an approach aims to identify RPL subgroups that are most likely to benefit from progesterone therapy.

This review synthesizes current evidence on the efficacy of progesterone in treating RPL, explores its underlying mechanisms, and highlights precision medicine approaches to optimize therapeutic outcomes. By establishing patient stratification and personalized treatment strategies, we aim to improve live birth rates, alleviate the multifaceted burden of RPL, and guide future management of this complex syndrome.

## 2 Progesterone for RPL: efficacy controversy and pharmacological rethinking

Early observational studies and some randomized controlled trials (RCTs) suggested that progesterone could significantly improve pregnancy outcomes in RPL patients, providing preliminary support for its clinical application (Daya et al., 1988; El-Zibdeh, 2005; Szekeres-Bartho and Balasch, 2008). However, these early studies were often constrained by small sample sizes and methodological limitations, which limited the reliability of their conclusions.

In recent years, several large-scale, high-quality RCTs and retrospective studies have yielded conflicting results (Table 1). The 2015 PROMISE trial, conducted in an unselected population with URPL (n = 836), found that vaginal micronized progesterone (400 mg twice daily) did not significantly increase the overall live birth rate compared to placebo (Coomarasamy et al., 2016). In contrast, the larger 2020 PRISM trial (n = 4,153), while showing only a modest absolute increase in live birth rate (3%) in the overall population, demonstrated a significant benefit (absolute increase of 15%) in the subgroup of women with three or more prior miscarriages (Coomarasamy et al., 2020). Another high-quality RCT published in 2023 failed to show a significant improvement in live birth rate with progesterone in women with one or more prior pregnancy losses (McLindon et al., 2023). Furthermore, a large 2025 retrospective study indicated that while univariate analysis showed no significant effect of oral dydrogesterone on live birth rate, multivariate analysis adjusting for factors such as maternal age, miscarriage frequency, body mass index and antiphospholipid syndrome revealed a statistically significant therapeutic benefit (Bashiri et al., 2023).

**TABLE 1:** Summary of key clinical trials and studies on progesterone therapy for recurrent pregnancy loss.

Study (author, year)	Study design	Population characteristics	Intervention (progesterone group)	Control group	Primary outcome (LBR ≥24 weeks)	Key findings and interpretation
Coomarasamy et al., 2016 (PROMISE)	Multicentre, double-blind RCT	Women (18–39 years old) with URPL (≥3 first-trimester losses)	Vaginal micronized progesterone (natural), 400 mg twice daily (N = 404)	Vaginal placebo capsules, twice daily (N = 432)	65.8% vs. 63.3%; RR = 1.04 (95% CI: 0.94–1.15)	No significant benefit. The study did not find a statistically significant increase in LBR in an unselected URPL population
Coomarasamy et al., 2020 (PRISM)	Multicentre, double-blind RCT	Women (16–39 years old) with early pregnancy bleeding Subgroup: ≥3 prior miscarriages	Vaginal micronized progesterone (natural), 400 mg twice daily (N = 2,079)	Vaginal placebo capsules, twice daily (N = 2,074)	Overall: 75% vs. 72%, RR = 1.03 (95% CI: 1.00–1.07) Subgroup (≥3 losses): 72% vs. 57%, RR = 1.28 (95% CI: 1.08–1.51)	Significant benefit in high-risk subgroup. While the overall effect was modest, women with a history of ≥3 miscarriages showed a clinically meaningful and significant increase in LBR.
McLindon et al., 2023 (STOP)	Double-blind RCT	Pregnant women (<10 weeks) with threatened miscarriage	Vaginal micronized progesterone (natural), 400 mg nightly (N = 139)	Vaginal placebo capsules, nightly (N = 139)	Overall: 82.4% vs. 84.2%, RR = 0.98 (95% CI: 0.88–1.09) Subgroup (≥1 losses): 80.6% vs. 84.4%, RR = 0.95 (95% CI 0.82–1.11)	No significant benefit. Progesterone did not improve LBR in women with threatened miscarriage, including those with a history of prior loss
Bashiri et al. (2023)	Retrospective cohort study	Patients with a history of RPL	Oral dydrogesterone (synthetic), 10 mg twice daily (N = 509)	No progesterone treatment (N = 357)	Unadjusted analysis: 80.6% vs. 84.0%, OR = 0.787 Adjusted analysis: Significantly higher, OR = 1.592 (95%CI: 1.051–2.413)	Potential benefit after adjustment. Univariate analysis showed no difference, but after adjusting for confounders (e.g., age, miscarriage frequency, body mass index and antiphospholipid syndrome), dydrogesterone was associated with significantly higher LBR.

Abbreviations: CI, confident interval; LBR, live birth rate; OR, odds ratio; RCT, randomized controlled trial; RPL, recurrent pregnancy loss; RR, risk ratio; URPL, unexplained recurrent pregnancy loss; vs., versus.

Meta-analyses on this topic have also yielded inconsistent conclusions. Two analyses published in 2015 and 2017 suggested progesterone reduced miscarriage rates, their findings are compromised as both included a RCT that was subsequently retracted due to data integrity concerns (Carp, 2015; Saccone et al., 2017). A 2024 meta-analysis of three RCTs (including the PRISM trial) concluded progesterone increased live birth rates with low heterogeneity, but the certainty of evidence was rated as moderate (Zhao et al., 2024). Conversely, a 2025 Cochrane review incorporating nine controlled trials found that progesterone had no significant effect on miscarriage or live birth rates in URPL (Haas et al., 2025).

Briefly, while early research and some subgroup analyses suggest that progesterone may have a potential benefit in RPL patients, results from high-quality RCTs and meta-analyses remain contradictory. Current evidence is insufficient to widely recommend progesterone for all women with RPL. These conflicting findings highlight a fundamental flaw in traditional trial design: the “one-size-fits-all” approach that asks, “Is progesterone effective for all RPL patients?” This design ignores the profound molecular heterogeneity underlying RPL, diluting treatment effects by enrolling mechanistically diverse populations. The PRISM subgroup analysis sends a clear signal that the critical question is instead: “For which RPL patients with specific biological characteristics is progesterone effective?” Therefore, there is an urgent need for precise screening and stratification of patients to validate the efficacy of progesterone and clarify its appropriate clinical application. The key to achieving this goal lies in exploring the association between the mechanism of progesterone action and the pathogenesis of RPL, thereby accurately identifying the population that would benefit from progesterone therapy.

## 3 The mechanisms of progesterone: linking to the pathogenesis of RPL

The therapeutic efficacy of progesterone is highly dependent on its multi-faceted physiological mechanisms, which correspond to different molecular defect phenotypes among RPL patients. Understanding this correspondence is fundamental to patient stratification.

### 3.1 Endometrial receptivity regulation and the “receptivity-defective” subtype

Upon conception, progesterone activates the *PR* in endometrial stromal cells, inducing decidualization to facilitate embryo implantation (Kasid and Laumas, 1981; Patel et al., 2015; Tan et al., 1999). This transient and precisely regulated process is known as the window of implantation (WOI). Studies have shown that insufficient progesterone levels or resistance to progesterone in decidual cells, due to factors such as inadequate clearance of senescent decidual cells (DCs), can lead to a shortened luteal phase and impaired endometrial development (Craciunas et al., 2021; Günther et al., 2023; Lucas et al., 2020). This may result in a displaced WOI (either advanced or delayed), ultimately contributing to RPL. Furthermore, aberrant gene expression profiles have been observed in the endometrium of some RPL patients, including dysregulation of receptivity-associated genes such as *LAMB3*, *HOXA10*, *MUC1*, *PROK1*, *LIF*, and *ITGB3* (Achache and Revel, 2006; Karaer et al., 2014; Meseguer et al., 1998; Pearson-Farr et al., 2022; Xu et al., 2012). These abnormalities point to impairments in downstream progesterone signaling pathways. For this “receptivity-defective” RPL subtype, timely and adequate progesterone supplementation may help restore or widen the implantation window (Labarta et al., 2021).

### 3.2 Immunomodulation at the maternal-fetal interface and the “immune-dysregulated” subtype

Immune homeostasis at the maternal-fetal interface is crucial for sustaining pregnancy. A considerable proportion of RPL cases present an “immune-dysregulated” phenotype, characterized by aberrations in key immune cell populations that disrupt the precisely balanced tolerogenic environment necessary for embryo implantation and development. The therapeutic potential of progesterone in RPL is profoundly linked to its ability to modulate and correct these specific immune disturbances.

#### 3.2.1 Uterine natural killer (uNK) cell dysfunction

In normal pregnancy, progesterone signaling through the *PR* upregulates interleukin-15 (IL-15) in the endometrial stromal cells, which promotes the proliferation and functional maturation of uNK cells (Alecsandru et al., 2021; Strunz et al., 2021; Wilkens et al., 2013). These cells increase during early pregnancy and subsequently decrease, playing crucial roles in vascular remodeling and immune regulation. In contrast, uNK cell dysfunction is observed in many RPL patients, often characterized by either abnormally elevated and persistent cell numbers or increased cytotoxic activity (Tang et al., 2011; Trundley and Moffett, 2004). This dysregulation disrupts local immune balance, impairing trophoblast invasion and placental development. Progesterone can modulate the cytotoxic profile of uNK cell and promotes their switch to a more regulatory, cytokine-secreting phenotype (Shah et al., 2018).

#### 3.2.2 Treg/Th cell imbalance

Successful implantation is associated with a shift from a pro-inflammatory state dominated by Th1 and Th17 to an anti-inflammatory, tolerogenic state dominated by Th2 (Garmendia et al., 2025; Wang et al., 2020). In addition, Treg cells are also vital for suppressing deleterious maternal immune responses against the semi-allogeneic fetus (Jameel et al., 2024). However, in RPL, this immune balance is often skewed toward inflammation. Patients frequently show significantly decreased percentages of Th2 and Treg cells in both peripheral blood and decidual tissue, alongside elevated proportions of pro-inflammatory Th1 and Th17 cells, contributing to implantation failure and early pregnancy loss (Abdolmohammadi Vahid et al., 2019; Kwak-Kim et al., 2003). Progesterone can counteract this inflammatory shift by promoting a Th2-biased response and serving as a potent inducer of Treg cell differentiation and expansion (Areia et al., 2015; Piccinni, 2006).

#### 3.2.3 Macrophage polarization aberration

Decidual macrophages can polarize into two main functional phenotypes: the classic pro-inflammatory M1 type, induced by Th1 cytokines like IFN-γ and TNF-α, and the alternative anti-inflammatory M2 type, promoted by Th2 cytokines like IL-4 and IL-13 (Gordon and Martinez, 2010; Wang et al., 2014). A dynamic balance, tilting towards an M2-dominant profile, is essential for maintaining tolerance, supporting tissue repair, and facilitating trophoblast invasion. In RPL, this balance is broken, with an overactivation of M1 macrophages and a suppression of M2 functionality. This polarized imbalance contributes to a pro-inflammatory microenvironment, inhibits extravillous trophoblast proliferation and invasion, and ultimately compromises pregnancy maintenance (Wang et al., 2022; Yang et al., 2022). Progesterone signaling promotes the anti-inflammatory M2 phenotype, thereby helping to restore the critical M1/M2 balance at the implantation site (Gordon and Martinez, 2010).

Therefore, for RPL patients with underlying immune dysregulation, progesterone therapy is not merely hormonal support but a targeted immunomodulatory intervention. By simultaneously regulating uNK cell function, restoring the Treg/Th17 balance, and optimizing macrophage polarization, progesterone can recalibrate the immune landscape at the maternal-fetal interface. This shift from a state of inflammation and rejection towards one of tolerance and acceptance provides a strong mechanistic rationale for using progesterone in the “immune-dysregulated” RPL subset, offering a pathway to re-establish the immune homeostasis which is necessary for a successful pregnancy.

### 3.3 Uterine function regulation and other potential subtypes

Progesterone plays a crucial role in maintaining uterine function and promoting placental vascular development during early pregnancy. By activating calcium channels mediated by the *PR* on uterine smooth muscle cells, it effectively inhibits excessive uterine contractions (Maliqueo et al., 2016). Simultaneously, progesterone upregulates the expression of key angiogenic factors such as vascular endothelial growth factor and placental growth factor (Park et al., 2020). Together, these actions promote the remodeling of uterine spiral arteries, enhances placental blood perfusion, and thereby reduces the risk of miscarriage in early gestation.

It is noteworthy that inadequate vascular network formation and insufficient placental circulation are closely associated with the etiology of RPL in some patients (Aguilar and Mitchell, 2010). In this context, the role of progesterone becomes particularly significant, as it can substantially improve placental blood supply through the aforementioned mechanisms, thereby providing essential support for the maintenance of pregnancy.

## 4 Patient stratification strategies: a precision pharmacology perspective

**FIGURE 1:**
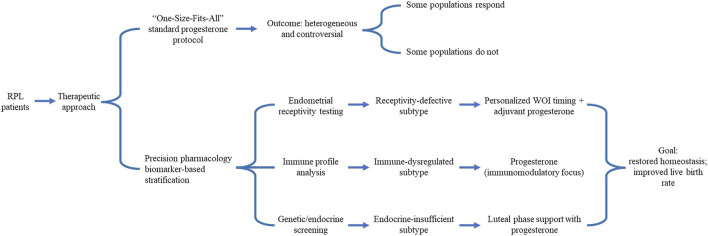
Contrasting two paradigms of progesterone treatment in RPL: From a “One-Size-Fits-All” approach to precision pharmacology-based stratification. Abbreviations: RPL, Recurrent pregnancy loss; WOI, Window of implantation.

The controversy over progesterone efficacy largely reflects profound heterogeneity among RPL patients. Recent studies increasingly stratify patients by molecular or immunological profiles to identify responders, enabling precision progesterone therapy (Figure 1). Although valuable, this framework simplifies clinical reality; because overlapping abnormalities frequently coexist, this targeted approach warrants clinical caution.

### 4.1 Stratification based on clinical features

#### 4.1.1 Number of previous miscarriages

The PRISM trial subgroup analysis showed that women with three or more prior miscarriages experienced a significantly higher live birth rate following progesterone therapy, a benefit not observed in women with fewer losses. This suggests that a higher frequency of miscarriage may correlate with greater responsiveness to progesterone.

#### 4.1.2 Endocrine profile

A classic subgroup likely to benefit from progesterone supplementation consists of RPL patients with biochemical indicators of subclinical luteal phase deficiency-for example, low mid-luteal serum progesterone levels (Daya et al., 1988; Pluchino et al., 2014).

### 4.2 Stratification based on molecular biomarkers

#### 4.2.1 Endometrial receptivity markers

The Endometrial Receptivity Array (ERA) can identify a displaced WOI, while transcriptomic analysis may detect specific gene expression signatures; both approaches can help select “receptivity-defective” patients (Díaz-Gimeno et al., 2013; Liu et al., 2022).

#### 4.2.2 Immune cell profiles

Immunohistochemical or flow cytometric analysis of endometrial biopsies can quantify uNK cell density and Treg cell proportions. “Immune-dysregulated” patients with high uNK cell cytotoxicity or low Treg proportion may be more sensitive to the immunomodulatory effects of progesterone (Choi et al., 2000).

#### 4.2.3 Progesterone receptor and genetic variants

*PR* has two isoforms that result from alternative splicing events and give rise to *PR-A* and *PR-B*. Unlike *PR-A*, *PR-B* contains an additional 165 amino acids in its N-terminus that confers it with a unique transactivation domain (Conneely et al., 2001; Conneely and Jericevic, 2002). Current evidence indicates that the relative ratio of *PR-A* to *PR-B* is crucial for maintaining pregnancy, with an increased ratio in the myometrium being necessary for the initiation of parturition (Mesiano, 2004). Research demonstrates that *PR-B* is the predominant isoform throughout gestation, functioning to maintain myometrial quiescence, whereas *PR-A* promotes uterine contraction via pro-inflammatory mechanisms. Therefore, both the expression levels of *PR* and the ratio of its isoforms are key factors influencing endometrial responsiveness to progesterone (Peavey et al., 2021; Wu and DeMayo, 2017). Furthermore, single nucleotide polymorphisms in the *PR* gene that are associated with RPL risk could serve as potential genetic markers for predicting progesterone response (Bahia et al., 2018; Chiu et al., 1996).

### 4.3 Future directions: integrating multi-omics data

Future research should focus on integrating genomic, transcriptomic, proteomic, and metabolomic data to construct a molecular classification system for RPL (e.g., “immune,” “receptive,” and “vascular” subtypes). Such a system would enable the matching of optimal treatment strategies to each subtype, with progesterone serving as a core therapy for patients in the “immune” subgroup and in certain “receptive” subgroups (Chen et al., 2021).

### 4.4 Safety data related to progesterone use

Beyond the focus on the efficacy of progesterone in treating RPL, its safety during pregnancy is equally crucial. Long-term, high-dose use of certain synthetic progestins has been associated with specific risks in non-pregnant populations. However, in the context of RPL treatment, progestogen administration is typically short-term and aims to achieve physiological levels. To date, multiple large-scale RCTs have found no significant increase in relevant cancer risks, nor an elevated risk of congenital heart disease or other new safety issues (Tournaye et al., 2017).

## 5 Challenges and future perspectives

Despite the promising prospects of precision stratification, several key challenges must be addressed:

- Standardization of biomarker assays: current detection methods for existing biomarkers lack uniformity, such as uNK cell counts or PR expression, hindering clinical translation and comparison across studies (Chen, L. et al., 2024).
- Lack of prospective validation: most stratification hypotheses originate from retrospective analyses or *post hoc* subgroup analyses. There is an urgent need for validation in prospective, biomarker-guided RCTs.
- Optimization of drug formulations and regimens: different progesterone formulations exhibit distinct pharmacokinetic, tissue distribution, efficacy and side effects, which should be carefully evaluated to identify the optimal regimen for personalized treatment.

Future research should prioritize the following directions:

- Conduct clinical trials that actively recruit potential responders based on predefined biomarkers.
- Utilize cutting-edge technologies like single-cell sequencing and spatial transcriptomics to deeply dissect the microenvironmental heterogeneity of RPL, discover novel stratification targets, and identify functional downstream biomarkers capable of specifically distinguishing between PR isoforms and quantifying their expression levels.
- Given the frequent coexistence of multiple abnormalities, future research must explore combination strategies of progesterone with other agents (e.g., low-dose aspirin, immunomodulators) to address overlapping pathological mechanisms.

## 6 Conclusion

The long-standing controversy over progesterone use in RPL management reflects an insufficient understanding of both disease heterogeneity and interindividual variation in treatment response. The future path is no longer empirical administration to all RPL patients but a shift towards precision pharmacology practice. By stratifying patients using clinical features and molecular biomarkers (e.g., endometrial gene signatures, immune cell characteristics, PR genetic variants), we can precisely identify the “progesterone-sensitive” RPL subgroup. This paradigm shift will advance RPL therapy from a “one-size-fits-all” model into a new era of individualized medication, ultimately offering more patients a realistic prospect of achieving a successful pregnancy.
